# A unique artificial intelligence-based tool for automated CBCT segmentation of mandibular incisive canal

**DOI:** 10.1259/dmfr.20230321

**Published:** 2023-10-23

**Authors:** Thanatchaporn Jindanil, Luiz Eduardo Marinho-Vieira, Sergio Lins de-Azevedo-Vaz, Reinhilde Jacobs

**Affiliations:** 1 Department of Imaging and Pathology, Faculty of Medicine, OMFS-IMPATH Research Group, KU Leuven, Leuven, Belgium; 2 Department of Oral Diagnosis, Division of Oral Radiology, Piracicaba Dental School, University of Campinas, Piracicaba, Brazil; 3 Dental Sciences Graduate Program, Federal University of Espírito Santo, Vitória, Brazil; 4 Department of Dental Medicine, Karolinska Institute, Stockholm, Sweden

**Keywords:** mandible, mandibular canal, mandibular incisive nerve, oral surgery, dental implant

## Abstract

**Objectives:**

To develop and validate a novel artificial intelligence (AI) tool for automated segmentation of mandibular incisive canal on cone beam computed tomography (CBCT) scans.

**Methods:**

After ethical approval, a data set of 200 CBCT scans were selected and categorized into training (160), validation (20), and test (20) sets. CBCT scans were imported into Virtual Patient Creator and ground truth for training and validation were manually segmented by three oral radiologists in multiplanar reconstructions. Intra- and interobserver analysis for human segmentation variability was performed on 20% of the data set. Segmentations were imported into Mimics for standardization. Resulting files were imported to 3-Matic for analysis using surface- and voxel-based methods. Evaluation metrics involved time efficiency, analysis metrics including Dice Similarity Coefficient (DSC), Intersection over Union (IoU), Root mean square error (RMSE), precision, recall, accuracy, and consistency. These values were calculated considering AI-based segmentation and refined-AI segmentation compared to manual segmentation.

**Results:**

Average time for AI-based segmentation, refined-AI segmentation and manual segmentation was 00:10, 08:09, and 47:18 (284-fold time reduction). AI-based segmentation showed mean values of DSC 0.873, IoU 0.775, RMSE 0.256 mm, precision 0.837 and recall 0.890 while refined-AI segmentation provided DSC 0.876, IoU 0.781, RMSE 0.267 mm, precision 0. 852 and recall 0.902 with the accuracy of 0.998 for both methods. The consistency was one for AI-based segmentation and 0.910 for manual segmentation.

**Conclusions:**

An innovative AI-tool for automated segmentation of mandibular incisive canal on CBCT scans was proofed to be accurate, time efficient, and highly consistent, serving pre-surgical planning.

## Introduction

The mandibular incisive nerve, the terminal branch of the inferior alveolar nerve, continues its path within the bone toward the anterior region and provides innervation to the anterior teeth.^1–4^ The surrounded anatomical structure poses complexity due to its small diameter and less corticalization compared to the inferior alveolar canal.^2,5^ While often overlooked in anatomy textbooks as a safe region, the incidence of neurological disturbance involving the mandibular incisive nerve can reach 33%, with approximately 8% resulting in permanent symptoms. Implant placement-related nerve injury is reported to be around 3%, but when considering permanent symptoms, this percentage can rise to 12%. This suggests that implant placement accounts for more than 75% of cases of permanent neurological disturbances,^6^ with pain, discomfort and sensory disturbances, seriously impacting the patient’s quality of life.^7,8^

The importance of careful treatment planning with radiographs for assessing bone volume, morphology, and neurovascular structures cannot be overstated in preventing injuries.^9^ Digital dentistry has taken the processes of diagnosis and treatment planning to new heights, becoming an integral part of advanced dental care nowadays.^10^ Artificial intelligence (AI) can play a significant role in assisting with this, as AI refers to computer systems that can replicate human abilities. In the field of oral healthcare, AI has already shown promise in tasks such as disease detection, classification, segmentation, treatment planning, prognosis assessment, and disease prediction.^11,12^ It offers advantages such as enhanced efficiency and consistency compared to manual methods. Among the different AI techniques, convolutional neural networks (CNNs) are commonly used for various applications, contributing to both diagnosis and treatment planning.^13^

Since 2020, research on AI has been booming, with a growing interest in AI-based anatomical segmentations using CNNs models such as for the mandibular canal.^14,15^ This trend in dental research involves the application of panoramic radiographs or cone beam computed tomography (CBCT), focusing on the segmentation of the main canal course or its variations such as the anterior looping.^14–19^ Though, all studies published limited the segmentation to the inferior alveolar canal, typically ending at the mental foramen. The importance of considering both the mandibular canal and its incisive canal extension in the segmentation is crucial for promoting safe, comprehensive pre-surgical treatment planning as well as prevention of the possible complications arising from nerve injuries. By incorporating AI to accurately segment the mandibular canal with incisive canal extension, clinicians can enhance their ability to plan and execute implant procedures with optimal precision and minimize the risk of nerve damage. The latter is unique and vital for the symphyseal area where clinicians often have difficulties in proper canal identification and where the number of reported post-implant nerve injuries is particularly high amongst elderly and partially or fully edentulous patients.^9^

Thus, the aim of this study is to develop and validate a novel AI tool based on a CNN architecture for automated segmentation of the mandibular canal with incisive canal extension in CBCT scans.

## Methods and materials

### Ethical criteria

This study was conducted in accordance with the World Medical Association’s Declaration of Helsinki on Medical Research. It was previously approved at the local Medical Ethics Committee under protocol number S57587, a retrospective data set for segmentation purposes which did not involve any experiments on humans or using human tissue samples. Furthermore, no patients were imaged specifically for the purpose of this study, and all patient data were anonymized.

### Sample selection

A pool of 200 CBCT scans acquired from three CBCT units, 3D Accuitomo 170 (J. Morita, Kyoto, Japan), Newtom VGI Evo (QR, Verona, Italy), and Scanora 3Dx (Soredex, Tuusula, Finland) were used as secondary data for this study (Table 1). All data of representative patients aged 10–86 years old were obtained from the radiology database of the UZ Leuven Hospital, Leuven, Belgium. The population, with the mean age of 46.22 ± 19.03, comprised 47% female and 53% male patients. Eligibility criteria were verified using Xero viewer (v 8.1.4.160, Agfa HealthCare NV, Mortsel, Belgium). Inclusion criteria were CBCT scans acquired with a voxel size of 0.30 mm or less, with adequate sharpness and detail. Only scans that displayed the incisive canal extending into canine region on both left and right sides were included. CBCT scans that exhibited insufficient image quality such as blurred images, and excessive metal-induced or movement artifacts were excluded from the study. Random selection and pseudonymization were carried out prior to annotation process. Additionally, scans from patients with bone fractures, anomalies, or lesions were also excluded. Image selection did not consider patient age, gender, ethnicity, or number of teeth present since the aimed pilot model could generalize and be applicable to a wide range of patients and clinical situations.

**Table 1.** Acquisition devices and corresponding parameters of the study’s database

CBCT devices	Voxel size µm	FOV mm x mm	Number of cases
Accuitomo 170	160	80 × 80	11
Accuitomo 170	200	80 × 80	1
Accuitomo 170	250	100 × 100	14
NewTom VGI evo	100	80 × 80	3
NewTom VGI evo	125	80 × 80	40
NewTom VGI evo	125	100 × 100	3
NewTom VGI evo	150	80 × 80	3
NewTom VGI evo	150	100 × 100	18
NewTom VGI evo	200	80 × 80	4
NewTom VGI evo	200	100 × 100	21
NewTom VGI evo	200	120 × 80	11
NewTom VGI evo	250	100 × 100	57
NewTom VGI evo	250	150 × 120	7
NewTom VGI evo	250	160 × 160	2
NewTom VGI evo	300	240 × 190	3
Scanora 3Dx	200	100 × 100	2

FOV, field of view.

The scans were divided into three subsets with random allocation for CNN model training (160 CBCT scans) and fitting to the labelled ground truth, a validation set (20 CBCT scans) to optimize and select the ideal model architecture, and a testing set (20 CBCT scans) to evaluate the model’s prediction compared to experts. All CBCT scans were exported in Digital Imaging Communication in Medicine (DICOM) format.

### Ground-truth labeling

CBCT scans were uploaded to Virtual Patient Creator (v 1.0.0, Relu BV, Leuven, Belgium), an online, user-interactive cloud-based platform that allowed segmentation. Ground truth of mandibular canal with incisive canal extension for training and validation was annotated by three oral and maxillofacial radiologists, one of whom had more than 15 years of experience in three-dimensional image analysis. The other two radiologists had 3 and 1 year of experience respectively. The nerve tracing training was performed using CS 3D imaging software (Carestream Health Inc, NY, USA) with nerve tracing tool, and the protocol was discussed before the annotation. The labeling was independently executed under supervision of experienced radiologist. In instances where uncertainty regarding the location of the canal arose, a senior oral and maxillofacial radiologist with over 30 years of experience in three-dimensional image analysis was consulted as an expert advisor.

The model was previously trained for mandibular canal segmentation until mental foramen.^17,18^ Incisive canal extension was segmented from the mental foramen region to its most anterior visible portion, using the nerve tool (selection of specific points within a nerve canal) in multiplanar reconstruction (MPR). The images were segmented in the online platform while simultaneously viewing the CBCT scans in cross-sectional slices using Xero viewer on a second screen (Barco NV, Kortrijk, Belgium) with 24.1-inch and a spatial resolution of 1920 × 1200 pixels, in order to assist with the precise location of the canal. The MPR and cross-sectional slices were used to evaluate accuracy of the segmentation. After initial segmentation, refinement was accomplished using the contour tool (adapt mode for making changes to the current segmentation or spine deviation mode allows for the selective adjustment of certain points) and the brush tools (utilized for painting or manually erasing with adjustable size and depth) to get more accurate and finer representation. During the segmentation process, zooming, brightness, and contrast adjustments could be performed in the online platform. Livewire tool (automated boundary detection feature that uses intensity values) was applied for image reconstruction.

Segmentations were then exported as standard triangle language (STL) files and imported into the Mimics software v. 3.0 (Materialise, Leuven, Belgium) to standardize the segmented file confined to the apex of canine with radiographic reference. Resulting STL files were imported to 3-Matic software v. 15.0 (Materialise, Leuven, Belgium) for further analysis using both surface- and voxel-based methods. The evaluation was carried out on mandibular incisive canal.

Intra- and interobserver analysis for human segmentation variability was performed on 20% of the data set. All oral and maxillofacial radiologists were required to perform a second segmentation of the mandibular incisive canal, with the task being completed 30 days after their initial segmentation.

### CNN model

The CNN model used for canal segmentation was developed based on 3D U-net architecture, a neural network design specifically intended for image segmentation. The model’s core framework encompassed an encoder for classification and a decoder for acquiring localized classification information.^20^ For this study, the model consisted of four encoding and three decoding blocks made up of two convolutions, followed by a rectified linear unit (ReLU) activation and group normalization with eight feature maps. All convolutions had a kernel size of 3 × 3 × 3, one stride, and one dilatation. A max pooling operation was applied after each encoder with kernel size of two in all dimensions.^21^ The U-net model was trained as a binary classifier with a binary cross-entropy loss function.

$$L_{BCE}=y_{n}\times log(p_{n})+(1-y_{n})\times log({1-p}_{n})$$

The training model was optimized with Adam optimizer with initial learning rate of 1e-4 and progressively lowered during training. Random spatial augmentation included random rotation uniformly sampled from −10 to 10 degrees, minor elastic deformations applied with a 10% probability, affine scaling ranging from 0.8 to 1.2 in all directions, and random cropping by taking a random crop inside the existing image with an allowable range of 60% of the image in each dimension. This augmentation was applied with a probability of 10%.

### Time efficiency

The time required to complete the segmentation was measured in minutes using 40 CBCT scans for each method. For AI-based segmentation, time was recorded starting from when the DICOM data were opened in AI tool until the algorithm automatically performed segmentation of the mandibular canal with incisive canal extension, produced the full-resolution binary segmentation result, as well as generated the STL file. The time required for refined-AI segmentation was recorded similarly to AI-based segmentation with subsequent manual refinement, if necessary. For manual segmentation, time was recorded starting from when the DICOM data were opened in Virtual Patient Creator until the generation of a complete STL file.

### Analysis metrics

Spatial overlap-based metrics derived from confusion matrix for a binary segmentation task with the variables of true-positive (TP), true-negative (TN), false-positive (FP) and false-negative (FN) values was used for the voxel-wise comparison between the ground truth (manual segmentation by specialist) and the predicted segmentation (AI-based segmentation).^17,18,22–24^ Firstly, the value of sice similarity coefficient (DSC) represents the amount of overlap or intersection between two segmented objects.^25^ In this case, it represents the agreement between the predicted segmentation and the ground truth. It is defined by the following equation:

$$DSC(A,B)=\frac{2\left|A\cap B\right|}{\left|A\right|+\left|B\right|}=\frac{2\times TP}{2\times TP+FP+FN}$$

Moreover, the value of intersection over union (IoU) which is the standard performance measure for object category segmentation problem, represents the overlap over union of two segmented object.^25,26^ For given object, it indicates the similarity between the predicted segmentation and the ground truth. It is defined by the following equation:

$$IoU(A,B)=\frac{\left|A\cap B\right|}{\left|A\cup B\right|}=\frac{TP}{TP+FP+FN}$$

For the error representation, root mean square error (RMSE), one of the most frequently used metrics to assess overall error of samples,^27^ shows the imperfections of the fit between two surfaces. For this purpose, it represents distance (X) between two closest points from two segmentations and can be calculated by the following equation:

$$RMSE(X)=\sqrt{\frac{1}{n}(x_{1}^{2}{+x}_{2}^{2}{+...+x}_{n}^{2})}$$

The precision and recall values assess the agreement between the identified oriented boundary edge elements. Precision is the ratio that represents the fraction of voxels predicted to belong to the volume of the ground truth.^28^ Both of these metrics are computed using an overlapping region. Recall, also known as sensitivity, is the portion of segmented voxels in the ground truth that are identified by the predicted segmentation.^29^ Precision and recall measures are determined by the following equations:

$$Precision=\frac{TP}{TP+FP}$$

$$Recall=\frac{TP}{TP+FN}$$

Accuracy is one of the most widely recognized evaluation metrics in statistics.^29^ It defines as the number of accurate predictions, which includes both correct positive and correct negative predictions, divided by the total number of predictions. For this particular object, it refers to the rate of correct segmentation in relation to all the segmentations observed.

$$Accuracy=\frac{TP+TN}{TP+TN+FP+FN}$$

### Consistency

The intraobserver analysis involved comparing the DSC values for the first and second segmentations performed by each radiologist. The interobserver analysis involved comparing the DSC values obtained from segmentations performed by three oral and maxillofacial radiologists. The consistency of the AI tool’s analysis involved comparing the STL files generated from uploading 20 scans twice to the platform.

## Results

### Time efficiency

The average time for AI-based segmentation, refined-AI segmentation and manual segmentation for mandibular incisive canal was 00:10, 08:09 and 47:18 minutes, respectively. This means a 284-fold time reduction for AI-based segmentation as compared to manual segmentation. The time required for manual segmentation ranged from a minimum of 20:57 minutes to a maximum of 110:00 minutes (almost 2 hours). In contrast, AI-based segmentation took a maximum of 00:21 minutes. For refined-AI segmentation and STL creation of the mandibular and incisive canal, the required time reached up to 10:30 minutes (Table 2).

**Table 2.** Evaluation of time efficiency

Time efficiency (minutes)	AI-based segmentation	Refined-AI segmentation	Manual segmentation
Mean	00:10	08:09	47:18
SD	<00:01	<00:01	00:02
Min	00:04	03:25	20:57
Max	00:21	10:30	110:00

AI, artificial intelligence.

Unit: Minutes.

### Analysis metrics

**Figure 1.**
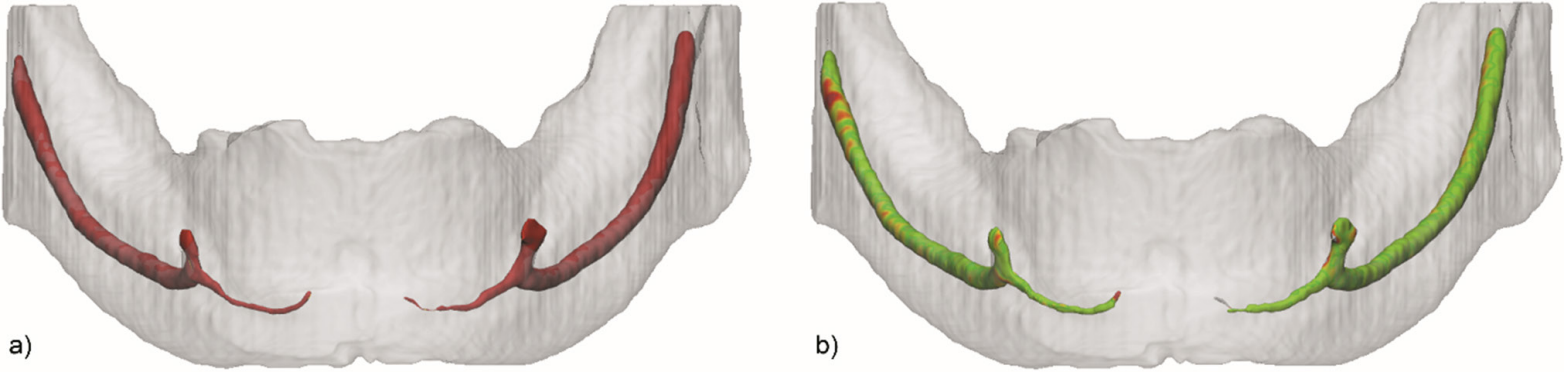
STL comparison map of mandibular with incisive canal extension. (a) Manual segmentation. (b) Automated segmentation with STL comparison map. STL, standard triangle language.

**Figure 2.**
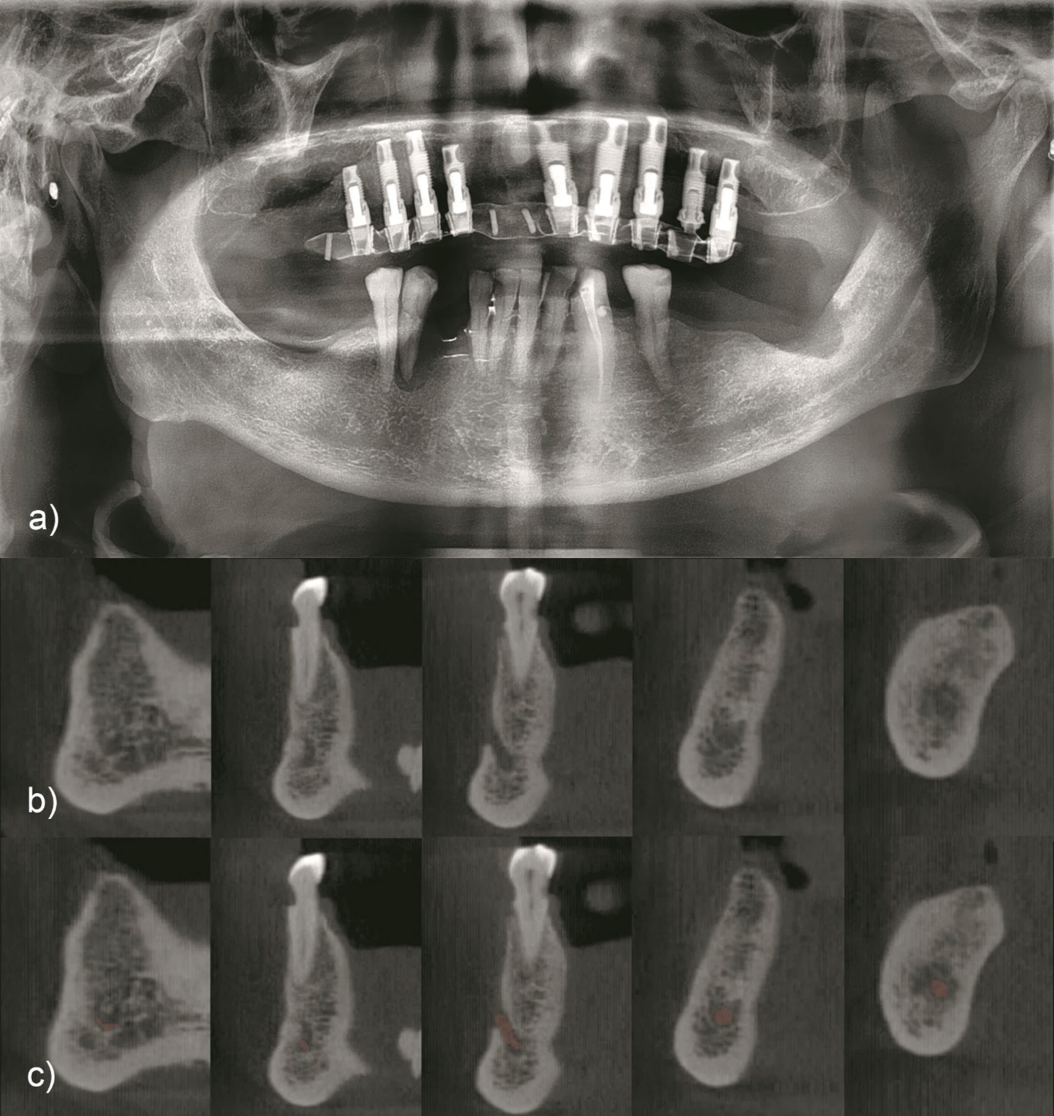
Case of 81-year-old male. (a) Panoramic radiograph. (b) Cross-sectional CBCT at incisor, canine, first premolar, second premolar and molar areas. (c) Cross-sectional CBCT with incisive and mandibular canal automated segmentation at incisor, canine, first premolar, second premolar and molar areas.

**Figure 3.**
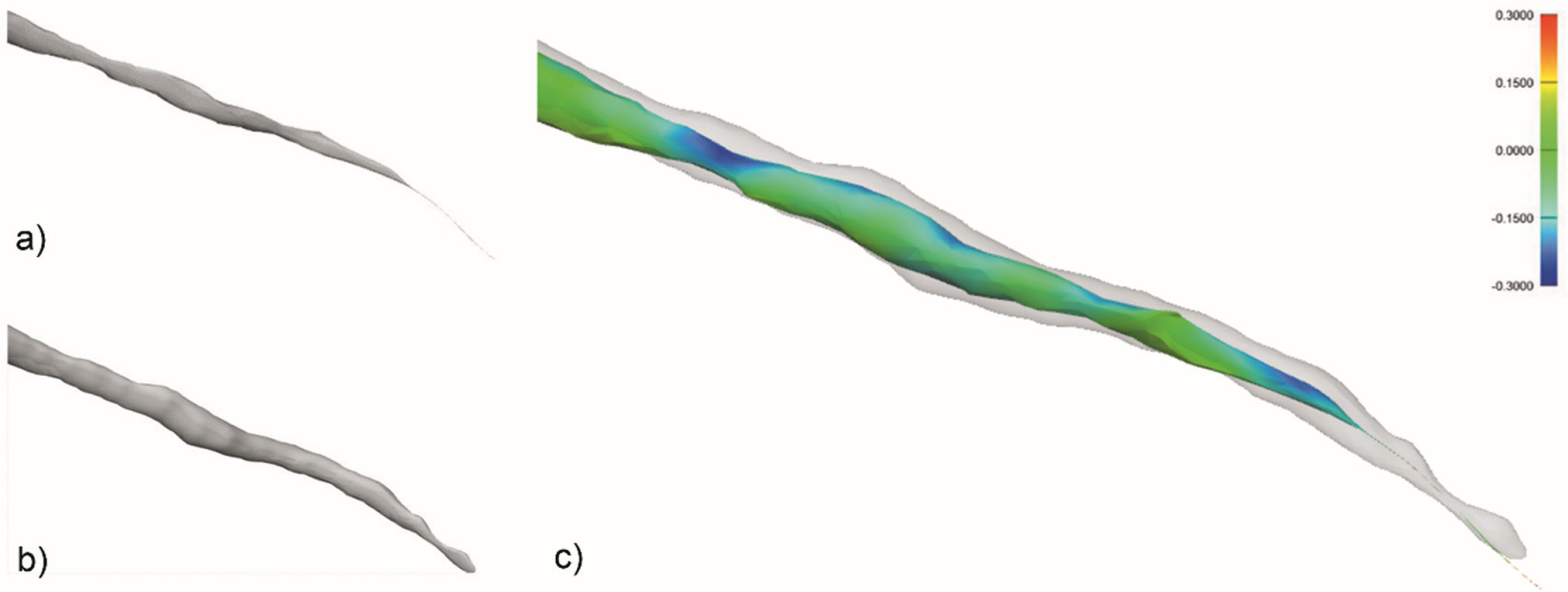
STL comparison map of incisive canal. (a) Manual segmentation. (b) Automated segmentation. (c) STL comparison map. STL, standard triangle language.

The metrics that indicate overlapping with manual segmentation showed that the AI-based segmentation provided DSC and IoU values of 0.873 and 0.775 respectively. On the other hand, the refined-AI segmentation showed a DSC of 0.876 and an IoU of 0.781. The minimum and maximum values of DSC and IoU for both methods exhibited a slight variation, with refined-AI segmentation demonstrating slightly higher values compared to AI-based segmentation. In terms of the imperfections in AI-based segmentation, the RMSE value was found to be 0.257 mm, while for refined-AI segmentation, the RMSE value was slightly higher at 0.267 mm. The RMSE values exhibited a relatively wide range, with refined-AI segmentation showing a larger gap compared to AI-based segmentation. The results showed a precision and recall of 0.837 and 0.890 for AI-based segmentation, and 0.852 and 0.902 for refined-AI segmentation. Both methods showed a minor variance in the minimum and maximum precision and recall values, with refined-AI segmentation also indicating slightly higher values than AI-based segmentation with an accuracy of 0.998 observed for both methods (Table 3, Figures 1–3).

**Table 3.** Evaluation of analysis metrics

Metrics	Descriptive analysis	AI-based segmentation *vs* Manual segmentation	Refined-AI segmentation *vs* Manual segmentation
DSC	Mean	0.873	0.876
	SD	0.025	0.028
	Min	0.827	0.828
	Max	0.927	0.933
IoU	Mean	0.775	0.781
	SD	0.041	0.045
	Min	0.705	0.706
	Max	0.864	0.874
RMSE	Mean	0.257	0.267
(mm)	SD	0.097	0.144
	Min	0.152	0.133
	Max	0.609	0.795
Precision	Mean	0.837	0.852
	SD	0.307	0.037
	Min	0.802	0.808
	Max	0.915	0.925
Recall	Mean	0.890	0.902
	SD	0.032	0.031
	Min	0.826	0.836
	Max	0.947	0.964
Accuracy	Mean	0.998	0.998
	SD	0.001	0.001
	Min	0.995	0.995
	Max	0.999	0.999

DSC: dice similarity coefficient, IoU: intersection over union, Max: maximum value, Min: minimum value, RMSE: root mean square error;SD: standard deviation.

### Consistency

**Figure 4.**
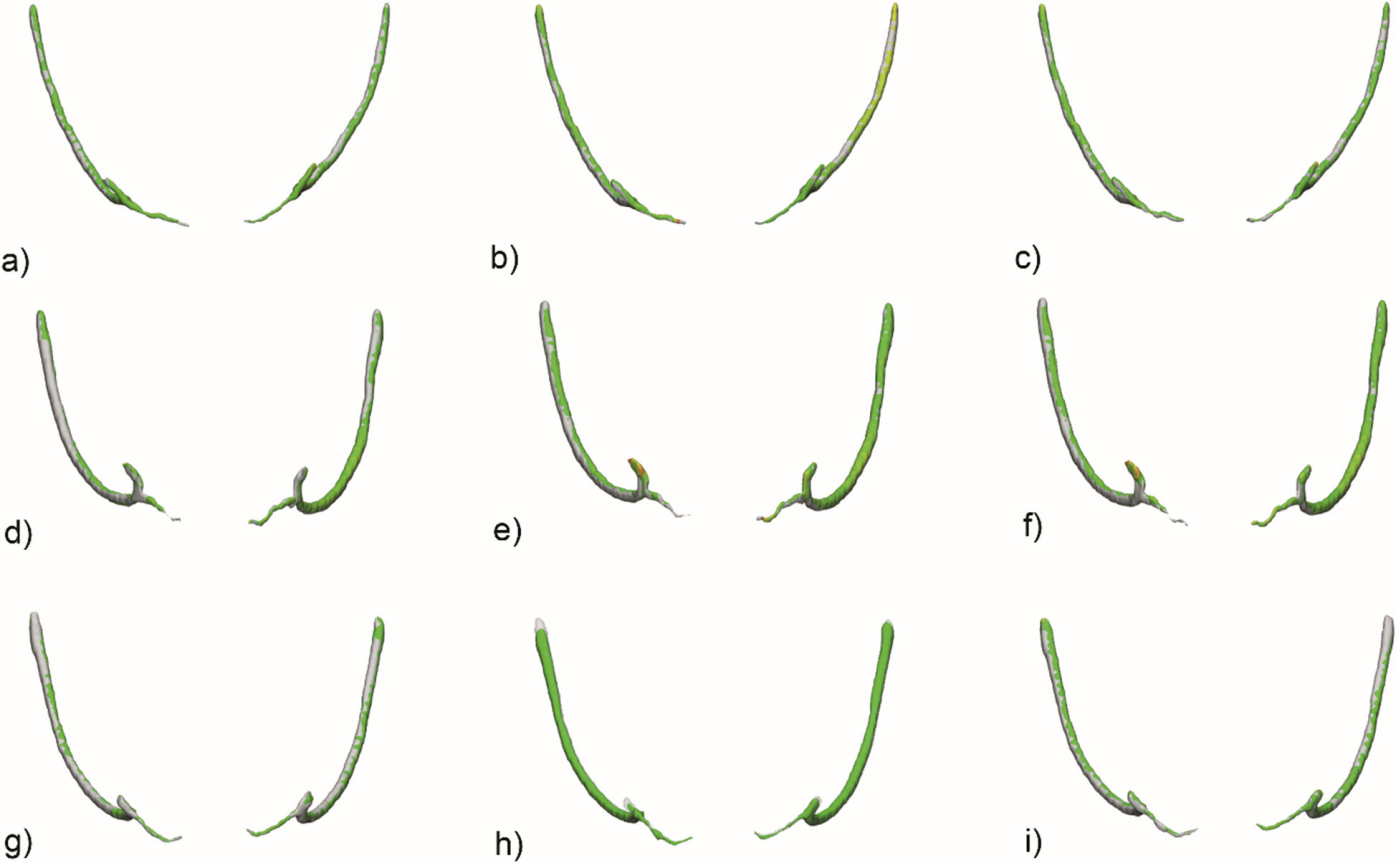
STL comparison map of mandibular canal with incisive canal extension between automated segmentation and observers. (a) Case 1 segmented by Observer 1. (b) Case 1 segmented by Observer 2. (c) Case 1 segmented by Observer 3. (d) Case 2 segmented by Observer 1. (e) Case 2 segmented by Observer 2. (f) Case 2 segmented by Observer 3. (g) Case 3 segmented by Observer 1. (h) Case 3 segmented by Observer 2. (i) Case 3 segmented by Observer 3.

The AI-based method’s consistency was determined using the DSC value, which signifies the degree of agreement between two segmentations, with a perfect consistency corresponding to a value of 1. On the other hand, the human observers' consistency exhibited an average agreement of 0.910 for intraobserver. Each observer contributed values of 0.914, 0.912, and 0.904, respectively. For interobserver analysis, the obtained value was 0.902 (Table 4, Figure 4).

**Table 4.** Agreement of observers

Agreement	Observer 1	Observer 2	Observer 3
Observer 1	**0.914**	0.890	0.893
Observer 2		**0.912**	0.922
Observer 3			**0.904**

**Bold:** Intraobserver analysis.

## Discussion

The initial and most critical phase in digital dental workflow is the segmentation of anatomical structures. This project has demonstrated the effectiveness of the AI-based segmentation tool in the mandibular incisive canal delineation, making it the most advanced tool known to us at present. Its uniqueness lies in its ability to segment complex structures such as the incisive canal extension alongside the mandibular canal.

Time required for segmentation performed in this study is the most significant parameter which ideally serve the purpose of digital workflow and significantly reduce tedious and time-consuming task of manually tracing and segmenting this delicate structure. This study revealed that AI-based segmentation was 284 times faster compared to manual segmentation. Taking into account the post-AI refinement process, which simulates the practitioner’s verification step before clinical application, the refined-AI segmentation continues to significantly save time compared to the manual approach on its own. Despite the challenges posed by the complexity and variability of the canal including the incisive canal extension, our findings align with previous studies on mandibular canal that have reported significantly faster for automatic segmentation compared to manual segmentation.^16,17^

In general, the evaluation metrics showed results varying from good to almost perfect for the entire mandibular canal. The DSC and IoU values depicting the segmentation overlap between AI-based and refined-AI methods demonstrated substantial agreement with manual segmentation. Nevertheless, since the values are not flawless, it is advisable for practitioners to review the structure before implementation. However, a wide range of RMSE values, especially with refined-AI segmentation, suggests that the additional manual refinement might introduce minor inaccuracies or variations in the segmentation, leading to a slightly higher RMSE value. Considering the potential for human bias and the uncertain improvements, one could question the justification of the additional time dedicated to refining AI.

The major difference has been influenced by the entire radiolucency canal detection of the AI compared to small delicate manual segmentation and a complication of the structure, despite the use of a precisely determined cut-off point based on reference anatomical structures from CBCT scans. However, the AI tool demonstrated high accuracy, signifying a substantial level of accurate prediction in alignment with the gold-standard, along with commendable precision and recall values. From a clinical perspective, a precise path with a substantially larger size of canal prediction ensured a safer pre-operative treatment plan to avoid neurovascular injury.

Well-known factors that have an impact on the quality of tomographic segmentation include image quality, the presence of artifacts, shape distortion, presence of pathology, object contrast, among others.^30–32^ Although variations in canal detection prevalence have been observed between sexes,^33^ model training using data obtained from three different machines with images regardless of age, sex, and ethnicity can lead to a generalized model. However, conducting a multicenter study would further enhance the generalizability of the data.

The radiologist expertise level has a direct correlation with the accuracy and consistency of radiographic segmentation. Although the utilization of advanced imaging modality can result in canal detection with a success rate over 90%,^2,34^ a degree of variation among the radiologists included in this study was noted. Anatomical aspects such as the density of canal cortex and density differences to the surrounding tissues are also relevant for canal detection.^35^ The thickness of the canal’s cortex was found to vary, with some areas having a complete cortex and others having no cortex.^22^ These various factors may have influenced the performance of both manual and automatic segmentation observed in this study, highlighting their significant impact on the segmentation outcomes.

Although the canal complexity, continuity, irregularity and size, AI-based segmentation showed a consistent smoothness, accurately defining the trajectory of the entire radiolucent space within the canal cortex and consistently stopped at the canine region even when the canal was visible further anterior, while manual segmentation appeared to be relatively small but also continuous, extending further anterior until the cortex could no longer be detected. Standardization with precise determination of the ending location is crucial for accurate analysis. From visual analysis, AI-based segmentation adhered strictly to the visual representation of the cortex which is also the similar challenges for practitioners in their daily practice, emphasizing the need for AI implementation to facilitate accurate treatment planning.

The results of previous studies indicated that AI tools demonstrated strong performance in the segmentation of maxillofacial structures.^17,23,24,36^ However, it should be noted that these structures were typically larger in size and had simpler anatomy, which made them easier to visualize in comparison to the mandibular and particularly the incisive canal. This inherent characteristic of the evaluated structures may have contributed to their better performance in comparison to the AI tool developed herein. When evaluating the model’s performance in comparison to other studies on the inferior alveolar nerve, this study achieved the highest DSC, IoU, and recall values, despite comprising the entire mandibular incisive canal segmentation. The precision value was slightly lower when compared to the study by Yang et al. (2023), which focused on panoramic radiographs. Notably, the accuracy matched that of studies conducted by Lahoud et al. (2022) and Kwak et al. (2020), all of which employed the U-net CNN model. When considering the segmentation time, this CNN model demonstrated the fastest performance compared to other studies on three-dimensional image segmentation.

Further improvement and evaluation of the AI tool is necessary to enhance its performance, especially with the generalization. In addition, it is recommended to conduct further studies that assess the performance of this AI tool on CBCT scans acquired under varying conditions, such as with different CBCT units and acquisition parameters, with anatomical variations, image artifacts, and pathological alterations, in order to evaluate its generalizability and applicability in routine clinical practice.

## Conclusion

An innovative AI-tool for automated segmentation of the mandibular canal with incisive canal extension on CBCT scans proofed to be accurate, time efficient, and highly consistent, serving pre-surgical planning. The findings of this research have the potential to improve pre-surgical planning procedures, as well as advancing the use of AI-powered automated segmentation in mandibular neurovascular canals.
